# Research Progress on the Role of Regulatory T Cell in Tumor Microenvironment in the Treatment of Breast Cancer

**DOI:** 10.3389/fonc.2021.766248

**Published:** 2021-11-15

**Authors:** Jianyu Liu, Xueying Wang, Yuhan Deng, Xin Yu, Hongbin Wang, Zhigao Li

**Affiliations:** Department of Surgical Oncology, Harbin Medical University Cancer Hospital, Harbin, China

**Keywords:** regulatory T cell, tumor microenvironment, breast cancer, immunotherapy, neoadjuvant treatment

## Abstract

The tumor microenvironment (TME) is a complex ecosystem comprised of cancer cells, stromal cells, and immune cells. Analysis of the composition of TME is essential to assess the prognosis of patients with breast cancer (BC) and the efficacy of different regimes. Treg plays a crucial role in the microenvironment of breast cancer subtypes, and its function contributes to the development and progression of BC by suppressing anti-tumor immunity directly or indirectly through multiple mechanisms. In addition, conventional treatments, such as anthracycline-based neoadjuvant chemotherapy, and neo-therapies, such as immune-checkpoint blockades, have a significant impact on the absence of Tregs in BC TME, thus gaining additional anti-tumor effect to some extent. Strikingly, Treg in BC TME revealed the predicted efficacy of some therapeutic strategies. All these results suggest that we can manipulate the abundance of Treg to achieve the ultimate effect of both conventional and novel treatments. In this review, we discuss new insights into the characteristics of Treg in BC TME, the impact of different regiments on Treg, and the possibilities of Treg as a predictive marker of efficacy for certain treatments.

## Background

In 1995, Sakaguchi et al. (1) described T cells (Tregs) as CD4+ CD25+ T cells with immunosuppressive effects on the human immune system. Tregs can suppress effector T cell responses as well as the activity of other immune cells, such as mast cells, dendritic cells, and B cells; thus, they are involved in cellular activation, maintenance of immune homeostasis (2), and allergy, while in malignant tumors they promote tumor progression by suppressing anti-tumor immunity (3, 4). The tumor microenvironment (TME) is a collective term for a complex ecosystem composed of heterogeneous cancer cells, stromal cells, and immune cells rather than a simple homogeneous population of cancer cells. Specifically, the immune cells in the TME consist of different cells, such as CD8+ CTLs CD4+ Th cells and Treg. However, the TME is relatively unique in different cancers. Among the TME of breast cancer (BC), tumor-infiltrating lymphocytes (TILs) are probably the most representative and studied component of BC and provide insights into the immunogenicity of breast cancers (5). However, when tumors are clinically detected, this immune response is, in most cases, unable to stop the cancer progression because tumors have developed the immune constructive process. Several studies have shown that, in primary breast cancer, Treg (6–8) infiltration of BC is associated with immune tolerance and leads to overall survival (OS) prognosis. Considering the important role of Treg in BC TME, it is necessary to evaluate the unique properties of Treg in BC TME by studying its onset, progression, and anti-immune mechanism. Many breast cancer drugs used today have also been shown to have direct or indirect effects on immunity, thus altering cancer progression. Therefore, we want to investigate the impact of these mechanisms on Treg. If these mechanisms can alter the abundance of Treg in BC TME, can Treg predict the effect of mechanisms, and can Treg abundance be used as a prognostic marker in BC patients? Here we will also discuss the latest advances in knowledge related to these questions.

## The Development of Treg in BC TME

**Figure 1:**
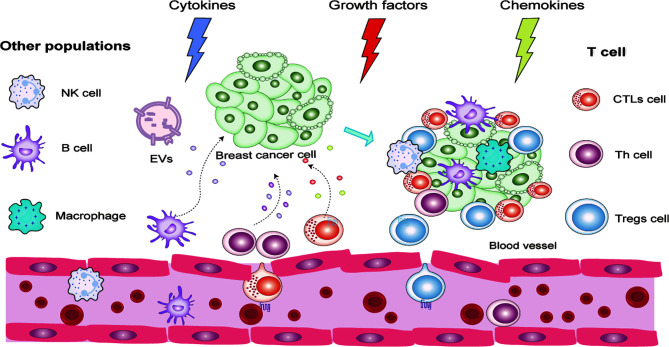
Graphic representation of the development of Tregs. The location of origin of Tregs consisted of the thymus and secondary lymphoid tissue. The process involved in the thymus includes the selection of high-affinity CD25+ Treg cells and the expression of FOXP3 and other essential receptors expressed on the membrane through a complex signal transduction. The other process taking place in the secondary lymphoid tissue is attributed to the binding of PD-1 and PD-L1 and the cytokine TGF-β, but the inner mechanism remains unclear.

Treg development begins in self-reactive thymocytes selected through high-affinity interactions with major histocompatibility complex (MHC) class II molecules expressed by thymic antigen-presenting cells (APCs) (4). A fraction of CD4+ CD8- thymocytes that receive strong T cell receptor (TCR) stimulation *via* self-antigen peptide–MHC complexes acquires the expression of CD25 (also known as IL-2Rα), which functions to increase the affinity for the interlukin-2 receptor (IL-2R) subunit CD122 (also known as IL-2Rβ). The IL-2–CD25 dimer then recruits CD122, followed by the common cytokine receptor γ-chain (γc). Subsequently, these three subunits make up a trimeric receptor expressed on Treg (9, 10). Upon IL2 and IL-2R binding, signaling occurs *via* multiple intracellular pathways, including the Janus kinase (JAK)–STAT pathway, the phosphoinositide 3-kinase (PI3K)–AKT pathway, and the mitogen-activated protein kinase pathway (11–13), wherein subsequent signaling *via* signal transducers and transcription activator 5 (STAT5) emits IL-2R signaling, leading to the expression of FOXP3, which confers various Treg cell-specific features to the cells, including the production of high levels of immune-suppressive molecules (14–16). In addition, signaling *via* the co-stimulatory receptor CD28 contributes to the commitment of a fraction of T cells in the thymus to the Treg cell lineage by inducing epigenetic and additional differentiation events in these cells (17–20). This commitment process involves many molecules; however, PI3K, AKT, and mTOR form a common intracellular signaling hub for TCR, CD28, and IL-2R that activates AKT through PI3K and mTOR complex 2, leading to the modulation of many cellular targets, including the forkhead box O family transcripts that are critical for Treg cell lineage commitment (21–23). As shown in **Figure 1**, we visualize the development process.

Thymus-derived Treg (tTreg) (formerly known as natural TREG—nTreg cells) and peripheral Treg (pTreg) cells (known as induced Treg—iTreg—cells when induced *in vitro*) are two types of Treg generated at different sites (4, 24). tTreg cells are generated through high-affinity contact with their own peptide MHC class II complexes in the thymus that are generated as a functionally mature T cell subpopulation. Under certain specific conditions, peripheral conventional T cells (Tconv) can differentiate into Treg cells in the presence of transforming growth factor-β (TGF-β) and are termed pTreg cells (25–31). However, whether this process requires the involvement of IL2 is unclear. Several studies supported the theories that IL-2 plays a key role in promoting TGF-β-mediated Foxp3^+^ expression in CD4^+^-naïve T cells, although it cannot induce Foxp3 alone (32–34).

**Figure 2:**
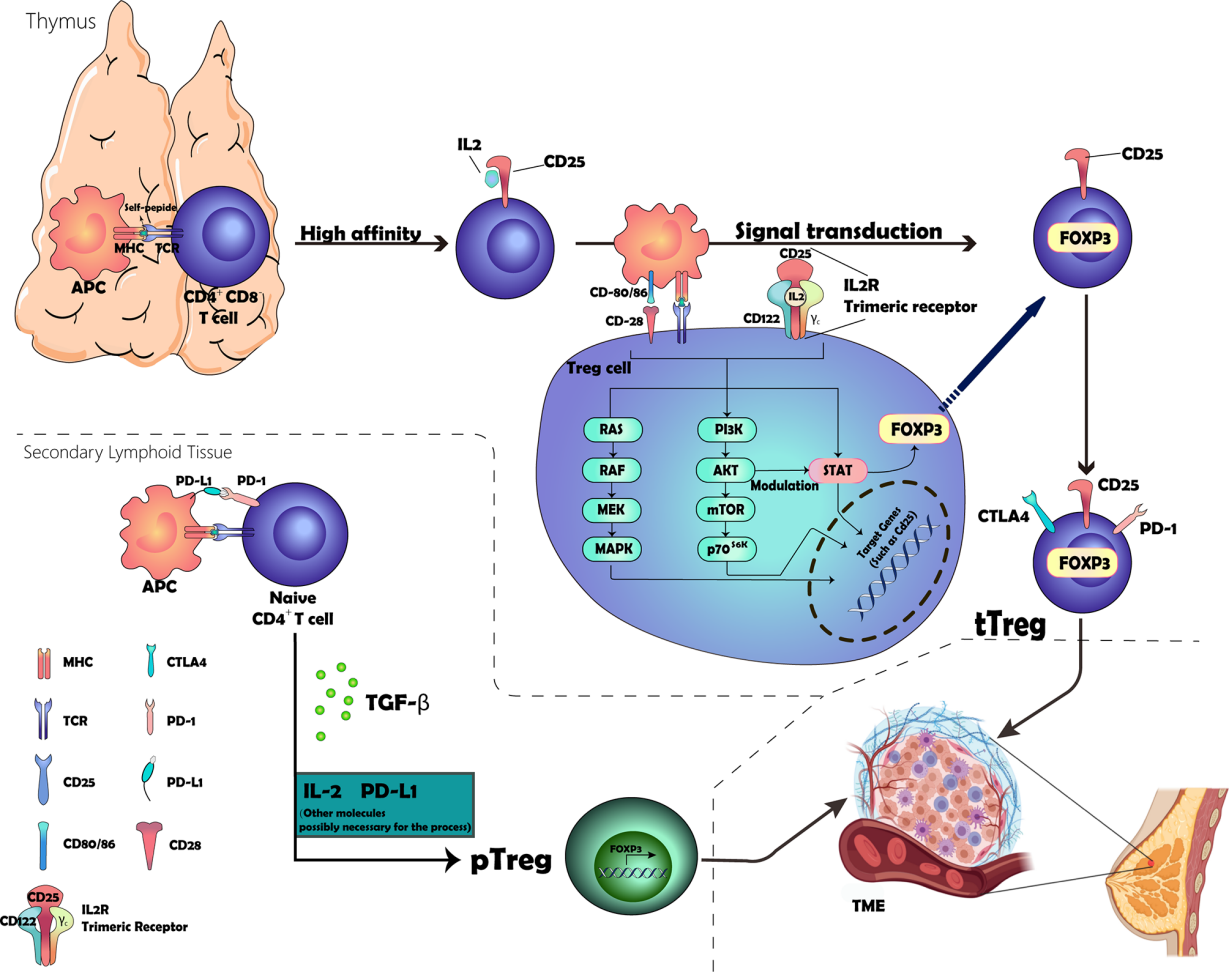
Detailed process of lymphocyte infiltration in a breast cancer microenvironment.

There is compelling evidence that PD-ligand 1 (PD-L1) plays a key role in the induction and maintenance of pTregs, leading to pTregs amplification in TME, which then inhibits T cell responses to tumors (35–38). *In vitro*, PD-L1 can induce Tregs in the absence of TGF-β, suggesting that PD-L1 signaling can promote pTreg development (36). *In vivo*, blocking PD-L1 signaling abrogates induction in a tumor-induced Treg transformation model even in the presence of TGF-β (39). The internal mechanism can be attributed to the reduction of the Akt signaling pathway, which is essential for pTreg cell development (40). The specific development and infiltration process of tTregs and pTregs are presented in **Figure 2**.

These two subgroups share similar phenotypic characteristics and suppressive function in response to T cell-mediated immune response and cancer. Although some minor differences are found between these two groups, such as mRNA transcript and protein expression, epigenetic modification, and stability, it is still difficult to distinguish them, so the term Tregs can, by default, be used directly to refer to FOXP3^+^ Tregs (41). Treg cells are chemo-attracted to the BC TME, where they can recognize their cognate antigens, be activated, and proliferate. The chemotaxis of Treg cells to the TME is mediated by combinations of chemokines and their receptors (for example, CCL22–CCR4, CCL28–CCR10, CXCL12–CXCR4, CCL5–CCR5, and/or CCL1–CCR8). They differ in different cancers (42–45). Especially in BC, CCR5, CCR8, CCR10, CX3CR1, CXCR3, and CXCR6 are stably and differentially expressed by tumor-resident Treg cells at the mRNA and protein levels (46, 47). While CCR4 was highly expressed by both tumor and peripheral blood Treg cells, CCR7 and CCR9 were downregulated in the Treg of TME. CCR5, CCR2, CXCR3, and CXCR6 were highly expressed by both tumor Tconv and Treg. However, CCR8 was found to be only highly enriched in tumor Treg cells and were much less abundant in Tconv cells, suggesting that Treg and Tconv cells may embrace both distinct and shared pathways to maintain their chemotaxis to the breast tumor microenvironment (47). In BC mouse models, blocking chemotactic signaling using antibodies or small molecules targeting CCL1–CCR8 reduces Treg cell accumulation in BC TME (46). Interestingly, these Treg cells recruit chemokines that can be produced not only by macrophages and tumor cells in TME (42–45, 47) but also by dysfunctional CD8+ T cells in TME that exhibit defective IL-2 production, such as CCL1 and CCL22.

Immune cell infiltrations are greatly heterogeneous between tumor types, and they can be located in different parts of the tumor, such as the center, margins, or adjacent lymphoid structures (48). High levels of Tregs in the periphery and TME were reported in peripheral and TME of breast (49), gastrointestinal tract (50), living carcinoma (51), pancreatic (52), and ovarian carcinoma (43). However, more Tregs infiltrate in TME than in adjacent normal tissue and peripheral blood in patients with primary breast cancer (47, 49, 53, 54). Notably, the density of Treg cells in the TME does not always correlate with matched peripheral blood (55). Within TME, Tregs were mainly distributed in the interstitial (also called mesenchymal) compartments and around the edges of BC infiltration (56). Interestingly, the specific TME in BC confers different characteristics to Treg cells. In a study by G Plitas et al. (47), the gene expression pattern of tumor-resident Treg resembled that of normal breast tissue but differed from that of corresponding activated or memory T cells isolated from peripheral blood, suggesting that the TME and its surrounding healthy regions are the main determinants of the gene expression characteristics of tumor and tissular Treg. TME usually contains large numbers of overexpressed immunosuppressive Treg cells of molecules, such as CTLA4 (57), PD-1 (58), LAG-3 (59), TIM-3 (60), and TIGIT (61), which are essential for their suppressive function (62). There have been many hypotheses on the composition of Treg in TME since Green et al. (63) who found amphiregulin to be expressed by Treg cells in a model of murine lung cancer. A more reliable conclusion is that Treg cells within TME in human cancer patients can be (i) tTreg recruited to the tumor site from outside the tissue and actively expanding (64) and/or (ii) a pool of pTreg derived from Tconv cells in periphery (64, 65) and/or, possibly, (iii) local expansion of tissue-resident Treg and/or (iv) Tregs converted from original TME- resident Tconv in TME. However, in BC TME, the difference of TCR sequence among blood and tumoral Tregs and Tconv cell was analyzed by Palita et al. (47). These analyses revealed a low TCR repertoire overlap between normal tissue and tumoral Treg cell and between intratumoral Treg and Tconv, which argue against hypotheses (iii) and (iv). However, both normal tissue and tumor Treg subsets contained large, expanded clones (47), similarly to the activated CD45RO+ (pTreg) but not to the resting CD45RA+ Tregs (tTreg) in peripheral blood, supporting hypothesis (ii) and denying hypothesis (i). These results together validated the possibility that, in breast cancer TME, the majority of Tregs in breast tumors were initially recruited from the periphery (lymphoid tissue and blood), after which their phenotypic and functional features were shaped by the local environment. On the other hand, in certain circumstances like the late stages of cancer progression, the Th1 cells may convert to Tregs, thus promoting cancer development and progression, consequently conferring negative prognostic effects on breast cancer patient outcomes (66, 67).

## Differences in TME Between Different Subtypes of BC

The TME of BC is relatively unique among the different subtypes. The immune infiltrate is heterogeneous and can be located in different parts of the tumor, such as the core (the center), the infiltrative margin, or the adjacent tertiary lymphoid structures. As for TILs, the most-studied component, it was higher in HER2+ and TNBC than in tubulointerstitial BC subtypes, as demonstrated by a secondary analysis of several clinical trials, such as FinHer (5), NeoALTTO (68), GeparQuattro (69), etc. Specifically, in a recent TNBC study (70), TME within TNBC is classified as immunoreactive subtype or “immune-cold” subtype by microdissection of tumor tissue. The CD8+ T levels are high, and PD-L1 was amplified, indicating a good effect of TME. However, in the “immune-cold” subtype, TME showed a negative expression of CD8+ T cells instead of the B7 family co-suppressor molecule B7-H4, which could suppress the effects of T cell effector function and infiltration. This result suggests that Her2-positive and Luminal BC can also be classified into subtype, and we can select the immune response subtypes for immunotherapy.

TME is diverse, but its signature is associated with primary cancer tissue, suggesting a link between BC and tissue-resident Tregs (71). Treg enrichment is thought to be reflected in more BC with a higher histological grade (47, 72), invasive characteristics of the tumor (73, 74), and BC subtypes (6, 56, 72). The Treg infiltration rate increases in the order of Luminal A < Luminal B < Luminal HER2 < HER2-enriched < basal-like breast cancer. TNBC had the highest proportion of CD4+ T cells among the subtypes of breast cancer, and thus Treg cells transformed by Tconv were particularly prominent. In addition, the higher number of Tregs in the HER2-enriched BCs is partly explained by the higher level of chemokines, cytokines (75, 76), and TGF-β (77, 78) present in TME. However, a recent study of Masanori Oshi overturned these theories (79). According to The Cancer Genome Atlas database, the abundance of Tregs in primary tumors was not related with BC subtype, American Joint Committee on Cancer staging, or Nottingham pathological grade. Strikingly, the Treg infiltrating order of subtypes was entirely consistent with the order in which the PD-L1 expression rate increased (72), indicating that chemokines, cytokines, and/or immune checkpoint may be the inner factor that determines Treg infiltration instead of these clinical characteristics. To date, besides *in vitro* or animal models, the correlation between PD-L1 expression in tumor cell and the amount of Tregs in TME has been evaluated in patients with gastric and colorectal carcinoma (80, 81). However, in BC, this correlation remains controversial. However, as mentioned above, basal-like breast cancer with a higher level of CD8+ T cells expresses amplified PD-L1 (70), so this correlation is likely to be present in all BC subtypes.

## The Role of Treg in the TME of Different BC Subtypes and the Establishment of Targeting Treg Treatment

There are several mechanisms Treg can perform to suppress immune cells (82), such as (i) releasing granzyme B and perforins to induce the apoptosis of effector cells (83), (ii) negative signaling to T cells through conversion of ATP to AMP, thereby inhibiting T cell proliferation and IL-2 formation (1, 84), (iii) interacting with B7 expressed by responder T cells through the CTLA-4 (85, 86), and (iv) secreting cytokines, IL-10 and IL-35, which are the key suppressive cytokines for Treg production to inhibit antitumor immunity and favor tumor growth by reducing effector expansion and cytokine production (IFNc and TNFa) (87). The effect of TGF-β1 on the generation of pTreg was well defined, but the suppressive function of TGF-β1 is still unknown. Three recent letters on TGF-β1 were published, two of which (88, 89) claimed that TGF-β1 did not work, but Stephen-Victor et al. (90) insisted that their debate can be attributed to the difference of gene editing. The conclusion from the study of Stephen can be attributed to the fact that they did not ablate the tgfb1 gene successfully but, rather, reverse it, which made the chromosomes fragile and triggered the mutant mice to death. Overall, Treg cells suppress strong antitumor immunity, thereby impeding an effective immune response to tumors. In addition to direct immunosuppressive activity, Treg cells can also inhibit the development of high endothelial venules by suppressing the self-amplification loop activated by mouse T cell (91, 92). Thus, the absence of Treg cell promoted the development of high endothelial venules, which have an important role in lymphocyte recruitment (91), representing a novel role of Treg cell in TME.

Considering these mechanisms of Treg action in TME, appropriate methods can be used to inhibit their anti-immunity effect. First of all, we can reduce the number of infiltrating Tregs while preserving the peripheral Treg—for example, anti-chemokines like anti-CCR4 mAb (93) and anti-CCR8 (94) treatments specifically depleted Treg in TME, with the result that Treg depletion will contribute to the activation of APC and upregulate CD80/86 expression to enhance the presentation of autoantigens and tumor antigens to Tconv cells, and these activated Tconv cells can then further activate the APCs. This positive loop inhibits anti-tumor immunity and inhibit tumor growth. It is worth noting that the CCR8 expression within Treg is exclusively on Treg cells in breast cancer (26), and the enrichment of CCR8 expression has been correlated with poor prognosis in patients with various types of cancer, including breast cancer and melanoma (47). Targeting CCR8 mAb may be a more effective therapeutic strategy than anti-CCR4 mAb.

In addition to anticancer factors, antagonizing cytokines that regulate Treg factors in TME may be another promising approach to inhibit Treg action—for example, TGF-β1 has a strong impact on pTreg production with insignificant immune-suppressive effect, so anti-TGF-β1 is highly likely to reduce Tregs. A study performed in melanoma has shown that the combination with anti-CTLA-4 mAbs and the TGF-β1 receptor serine/threonine kinase inhibitor galunisertib directly inhibited the generation of pTreg, increased the CTL/Treg ratio, and decreased the indoleamine 2,3-dioxygenase expression of APCs in tumor-draining lymph nodes (95). The fusion protein (M7824) combined by anti-PD-L1 and anti-TGF-β1 was also investigated in some studies (96), and M7824 exhibited a good effect in reducing Treg on patients with clinical benefits. Considering the suboptimal effect of anti-PD-1/PD-L1 or anti-CTLA-4 in the treatment of breast cancer, the addition of anti-TGF-β1 is still under investigation if it enhances the overall effect of improving anti-tumor immunity, and further studies are needed to evaluate Treg after using this drug.

In addition, it is even more important to inhibit Treg infiltration by targeting the molecules that perform the primary function—for example, anti-CTLA-4 is applied to stop the process of downregulating B7 expression on APCs. An increasing number of studies have shown the antibody-dependent cellular cytotoxicity (ADCC) effect of anti-CTLA-4 on Tregs based on this theory (97–99), while clinical responders of anti-CTLA-4-(ipilimumab)-treated melanoma patients can also achieve a depletion effect of Treg (100). Unlike CTLA-4, anti-PD-1 could not be included in our targeted Treg group despite the fact that it has been shown to be an effective option for treating cancer patients. This is because PD-1 is an auto-inhibitor of PD-1-expressing cells, and therefore inhibition of PD-1 in CD4^+^ T cell enhances the function of PD-1-expressing T cells and Treg cells (101), with the overall effect of increasing anti-tumor immunity. This phenomenon can be explained by the hypothesis that anti-programmed death-1 (PD1)/PD-L1 mainly targets PD-1^hi^ Tconv cells and has a greater effect on these cells than on Treg cells. Considering the characteristics of Tregs and the great differences between anti-Treg treatments, the anti-BC immunity strategy can be tailored to be an effective combination of immunotherapies and other targeted therapies.

## Treg Interactions With a Variety of Cells

TME provides an environment for residing Treg to interact with their other immune cells, fibroblasts as well as vascular endothelium in TME. The interaction between these cells in TME nurture direct contact or indirect signals that promote or inhibit breast cancer growth, invasion, angiogenesis, and metastasis.

The mutual communication between Treg and Tconv is mainly indirect. First of all, the CTLA-4 on Treg can capture its ligands CD80 and CD86 on APCs, thus impairing their ability for co-stimulation of Tconv cells (85, 102, 103). The loss of co-stimulation makes Tconv more vulnerable to Treg suppression, and these Tconv with high-affinity TCRs will die by apoptosis (104). In addition, the competition of Tconv against IL-2 and other cytokines (57, 84) and the conversion of ATP into AMP (1, 84) are other indirect reactions that prevent optimal T cell activation.

The high abundance of Treg was also associated with increased infiltration of M2 macrophages and T helper type 2 (Th2) cells and decreased infiltration of T helper 1 (Th1) cells (79, 105). Similarly, in one of our unpublished original papers, CIBERSORT algorithm was used to test the correlation between Treg and macrophages in BC. We found that Treg was positively related to macrophage 0 (M0) but negatively correlated with macrophage 1 (M1). The negative correlations between M1 and Treg can be attributed to the suppressing M1-to-Treg contact (106) and/or inhibiting effect of soluble factors like TNF secreted by M1 on the accumulation of Tregs in TME (107). Some studies have demonstrated that TNF produced by M1 can diminish the suppressive activity of Treg cells through the NF-κB pathway (108, 109).

Carcinoma-associated fibroblasts are abundant in TME and involve many cancerous features such as tumor cell proliferation, angiogenesis, drug resistance, and metastases (110, 111). In BC, their enhanced role in tumor invasion and metastases is more pronounced. In addition, cancer-associated fibroblasts (CAFs) are able to secrete chemokines and cytokines, such as TGFβ, CXCL12, VEGF, and IL6, which stimulate cancer cell proliferation, epithelial–mesenchymal transition, and migration (112–115). The interaction between Treg and fibroblast in TME is also well identified. In a study of Costa et al. (116), multicolor flow cytometry and principal component analysis were performed to classify CAFs into four subtypes. Notably, the most representative subtype, CAF-S1, characterized by a high expression of the six fibroblast markers (FAP, integrin b1/CD29, aSMA, S100-A4/FSP1, PDGFRb, and CAV1) except CAV1, was positively found to be correlated with the number and function of Tregs but negatively correlated with CD8+ T lymphocytes. The internal mechanism was also well studied, namely, that CAF-S1 secrets CXCL12, which attracts Tregs and retains these cells through OX40L, PL-L2, and JAM2. In addition, CAF-S1 increases T lymphocyte survival and promotes their differentiation into Tregs *via* B7H3, CD73, and DPP4.

The interaction between Treg and vascular endothelial cells is a two-way process. Vascular endothelial cells can lessen the infiltration of Treg through chemical signals and physical barriers; they can also downregulate Treg activity through the production of leptin (117). Correspondingly, Tregs have also been reported to reduce endothelial cell activity and their chemotaxis of T cells (118). First of all, adhesion molecules, such as intercellular adhesion molecule and vascular adhesion molecules, are two main factors that promote T cell infiltration (119, 120).

However, the vascular endothelium cannot upregulate the expression of these two molecules in TME, which leads to the difficulty of T cell penetration. Meanwhile, this low expression can be reversed prophylactically by Treg depletion (121), which can be another mechanism of anti-Treg treatment. Additionally, the vascular endothelium establishes a physical barrier that restricts T cell infiltration. Accordingly, the blockade of the VEGF–VEGFR2 axis reportedly inhibits tumor growth through the decreased recruitment of Treg cells in the BC TME of a preclinical mouse model (122). In gastric cancer, anti-VEGFR2 mAb ramucirumab has already shown to lessen the density of effector Tregs (eTregs) but preserve CD8+T cells in the TME (74). The clinical efficacy of the combination of anti-VEGF–VEGFR2 axis and immune checkpoint blockade has been found in NSCLC (123), gastric cancer (124), RCC (125), etc.

## The Heterogeneity of Tregs in Peripheral and TME

The heterogeneity of Tregs was generated during, before, and after the entry of Tregs into BC TME. When Tregs are in the periphery, it can be subdivided according to the difference of transcription factors. Under the appliance of the transcriptional factor FOXP3 and other two surface markers, CD25 and CD45RA, circulating Tregs can be divided into three main groups: fraction I—CD45RA^+^ CD25/FOXP3^lo^ naive Tregs, fraction II—CD45RA^+^CD25/FOXP3^hi^ eTregs, and fraction III—CD45RA^-^CD25/FOXP3^lo^ cells, non-Treg. Helios, another transcription factor from Ikaros family, expressed by Treg but not Tconv cells in mice (126), can further classify Treg cells. FrII Treg cells in human blood exclusively express Helios, while both Helios-positive and Helios-negative cells are included in Fr I and Fr III Helios^+^. It was proposed that the expression of Helios by human Treg cells may promote leukemic cell survival and angiogenesis in *in vitro* assays (127). Moreover, Helios-negative Tregs were found to have low levels of Treg-specific demethylation region demethylation, so it shows a higher inflammatory cytokine production (128) and lesser suppressive activity (129). Based on these characteristics of Helios in Treg, Helios represents an attractive target for cancer immunotherapy at present. Consistently, it was argued that agonistic anti-glucocorticoid-induced TNFR-related protein (GITR) antibodies could inhibit Helios expression in Treg cells, whereby executing its anti-tumor function (130). Besides Helios, other markers, like TIGIT, CD226 (128), CD15s, HLA-DR, TIM-3, CD177 (47), and ICOS (131, 132), are promising markers expressed by Tregs that have the potential to further classify Tregs based on their function.

Chemokines, such as CCR4, CCR6, CCR8, and CXCR3, have also been used to characterize peripheral Treg. In this review, attention was paid to CCR8 and CD177, which play the critical role exclusively in BC. The study of Plitas (47) has shown that CCR8 was significantly upregulated in intratumoral Treg cells compared with normal adjacent tissue residents and their peripheral counterparts. Obviously, the enrichment of CCR8 is also correlated with a worse prognosis in BC patients (47). Moreover, the ratio of CCR8 and Foxp3 mRNA amounts can be an independent prognostic factor for the survival of BC patients. CCL1 is a known cognate of CCR8 which is highly expressed by intratumoral myeloid cells (47). Stimulating CCL1 can also enhance the suppressive capacity of human Treg cells *in vitro* through the STAT3-dependent pathway (133). As a result, targeting CCR8+ Treg cells through anti-CCR8 mAb or anti-CCL1 neutralizing mAb provides an opportunity for the selective depletion of Treg cells as an immunotherapeutic approach for the treatment of breast cancer. CD177 is another protein associated with cell adhesion and migration, which is highly expressed by Treg cell subsets (10–50% of the total number of Treg cells in breast cancer) (47). The role of CD177 on Treg cells remains to be unclear, and it is very likely that CD177 performs some functions and further subdivides Treg. Compared with CCR8 expressed on all Treg cells, CD177 was found to be expressed highly on a subset of tumor-associated Treg cells through flow cytometry. Moreover, single-cell analyses confirmed that CD177 is expressed highly in some Treg clusters in BC TME (134)..

Upon entry, TME will also remodel Tregs, resulting in a high degree of heterogeneity in genomic, transcriptional programs and chemokine receptor expression within the tumor Tregs despite their strong similarity to effector molecules. Recent work using multiregional genome sequencing of tumors has revealed a high degree of tumoral subclonality difference between spatial regions (135), including breast cancer (136). As for differences in transcriptional programs, single-cell RNA-seq detected differences in the co-expression patterns between Treg subpopulations of checkpoint receptor genes (CTLA-4, TIGIT, and GITR and other co-receptors) in certain Treg subset that can be mutually exclusively expressed in other subsets, indicating a different spatial and functional distribution of these subpopulations. Considering the results mentioned above, it is critical to decipher the inner mechanisms that shape and stabilize the Treg cell phenotype through the whole process of Treg recruitment. This is essential for us to evaluate, *i*.*e*., the feasibility and safety of novel therapeutic approaches aiming at targeting a specific Treg target.

## The Relationship Between the Density of Treg and Prognostics of Patients With BC

As mentioned above, the abundance of Tregs in the TME is not always linked to those in matching peripheral blood, suggesting that the analysis of the TME where T cells directly interact with tumor cells is more essential in studies of cancer immunology. Interestingly, within the TME, the density of intratumoral and stromal Treg infiltration should be assessed separately because they are independent prognostic factors (137). In a study with 1,270 samples of whole-tissue sections, intratumoral infiltration by Tregs is highly correlated with the prognosis of breast cancer (6, 72, 138). Although stromal Treg is sensitive to chemotherapy, intratumoral Treg is a better prognostic predictor of patients with breast cancer (6, 139).

Survival analysis was conducted by some research teams with respect to Treg high- and low-density BC subgroups without considering the subtypes of BC. The mean DMFS, DFS, breast cancer-specific survival (BCSS), OS, and DSS were comparable between the two groups, so the Treg levels did not significantly affect DMFS, DFS, or BCSS (56, 79).. Then how about the correlation within each subtype?

In breast cancer, a high frequency of TILs is associated with poorer survival in patients with ER+ and Her2+ breast tumor (56), while in TNBCs, the most aggressive and immunogenic subtype (140–142), the high incidence of TILs is significantly associated with longer survival (79, 143–145), indicating that the mere presence of TILs is insufficient to precisely predict their influence, and disease progression and clinical outcomes are influenced by TIL subtypes and their biological and functional characteristics rather than their density (146). Bohling and Allison (147) found a possible association between Treg infiltrates with TNBC subtype. According to Joe Yeong (148, 149), patients with TNBC exhibiting high intratumoral Treg density also have significantly longer DFS and OS than those with fewer intratumoral Tregs. In addition, some studies have demonstrated the association between Tregs in TME with HR- and HER2+ (6, 137, 150, 151). Jiang et al. (152) found that an abundant Treg infiltrate had an opposing prognostic significance in HR- and HR+ BC. The prognostic significance of Tregs was associated with HR- tumor status. On the HR- BC subgroup, high Treg showed a favorable effect on BCSS, in contrast to the lack of impact on BCSS among HR+ BCs (56). However, M Gobert et al. and (53) and GJ Bates et al. (8) found that the abundance of Treg has an influence on prognosis in HR+ BCs, while the prognostic value is unfavorable. The relation between Treg and prognostic value in HER2 overexpression is also controversial. In BC patients where an association between Treg infiltration and HER2 overexpression was discovered, Tregs were mainly linked to poor prognostics, such as higher tumor grade and decreased OS and PFS (6, 150). In addition, Tsang et al. (153) found that TILs were associated with a smaller tumor size in HER2-enriched tumors. However, he considered both cytotoxic CD8+ T lymphocytes and Tregs together as a factor and observed only a correlation between this subtype and the CTL, which could explain why the TIL was associated with a better prognosis. As we have mentioned above, no statistically significant difference was found with respect to Treg in relation to tumor stage, lymph node status, and tumor size. Nonetheless, a lower CTL/Treg ratio was observed among locally advanced BCs as compared to early BCs (56). Moreover, the recruitment of Tregs to TME has been associated with the development of metastases in patients with BC (73, 74, 154–156).

Some immune checkpoints expressed on Tregs also have a certain prognostic value. CTLA-4, expressed on the surface of naive effector T cells and Tregs with a low level, was the first clinically targeted immune checkpoint molecule (157). CTLA-4 has a high affinity toward CD80 and CD86, thereby dampening the stimulatory signals and attenuating T cell activation by interrupting the conventional TCR signaling (158, 159). In the TME, CTLA-4 inhibits immune response and promotes tumor cell survival (159). CTLA-4+ tumor-infiltrating Tregs could also contribute to tumor immune evasion by suppressing antitumor immunity and downregulating CD80/86 expression on APCs (86). A higher expression of CTLA-4 on Tregs in BC TME compared to peripheral blood Treg cells revealed more active and proliferative Treg cells in TME (47). PD-1 and PD-L1 are expressed on the surface of both activated T cells and Tregs. PD-1 and its interactions with PD-L1 play important roles in the tumor evasion of immune responses through different mechanisms, including inhibition of effector T cell proliferation, reducing cytotoxic activity, induction of apoptosis in T cells, and Treg expansion in TME. As we have mentioned above, Treg infiltration is likely to be an unfavorable factor in the HR-positive and triple-negative BC patients. Interestingly, Li et al. (72) noticed that, in the TNBC, PD-L1 was also proved to be an independent unfavorable prognostic factor for OS by multivariate analysis adjusted by age, tumor size, grade, and lymph node status. However, there was nearly no data and study to specifically investigate the abundance of PD-1 and PD-L1 expressed on Treg in BC TME. Considering the unique function of Treg, further studies are warranted to analyze these two molecules on Treg using flow cytometry and other experimental methods. The treatment of breast cancer includes the treatment of local disease with surgery, radiation therapy, and systemic treatment with chemotherapy, endocrine therapy, biologic therapy, or combinations of these. In this section, we will introduce the latest information on the role of Treg in the systemic treatment of BC. We put a great emphasis on both the influence of different regimes on the density and function of Tregs and the impact of Treg on the efficacy of different treatments in preoperative stage. The efficacy marker of drugs or regimens includes pathological completed response (pCR), objective response rate, etc.

## The Correlation Between Tregs and Different Therapeutic Strategies of BC

### CDK4/6

Cyclin-dependent kinases 4 and 6 (CDK4/6) are fundamental drivers of the cell cycle and are required for the initiation and progression of various malignancies. The pharmacologic inhibitors of CDK4/6 have been found to have a significant activity against several solid tumors (160, 161). Their primary mechanism of action is thought to be the inhibition of phosphorylation of the retinoblastoma (RB) tumor suppressor, inducing G1 cell cycle arrest in tumor cells (162). Currently, three CDK4/6 inhibitors have now been approved by the FDA for the treatment of ER-positive metastatic breast cancer: palbociclib (PD0332991), ribociclib (LEE011), and abemaciclib (LY835219). S Goel et al. (163) used murine models of BC and other solid tumors to show that CDK4/6 inhibitors not only induce tumor cell cycle arrest but also promote anti-tumor immunity. Deng et al. (164) indicated that palbociclib or trilaciclib (another CDK4/6 inhibitor) significantly enhances Tconv cell activation, thus contributing to antitumor effects *in vivo*. However, in addition to the effect on Tconv cell, CDK4/6 can also markedly suppress Treg proliferation associated with the reduced activity of the E2F target, DNA methyltransferase 1 (DNMT1) (163). Similarly, in the studies of S Goel et al. (163) and JR Whittlee et al. (165), the flow cytometric analysis of breast cancer in murine revealed that abemaciclib or the combination of fulvestrant–palbociclib did not alter the fractions of most types of TIL but significantly increased the CD3+ T cells and reduced the Tregs in both the TME and periphery. Moreover, the CTL/Treg cell ratio increased significantly in abemaciclib-treated tumors, further suggesting a tipping of the immune balance in favor of anti-tumor immunity (163). In particular, the Treg was more sensitive to CDK4/6 inhibitors compared with other lymphocytes, and this behavior has been related to the high expression in these cells of the proteins of the CDK4/6–cyclin D-RB axis (166, 167) or the reduced activity of DNMT1 (163). Reduced expression of the immune checkpoint receptor PD1 on Tregs was also observed in the study of S Goel, which was consistent with the diminishment of the immune-suppressing function of Treg in BC TME (163), suggesting that CDK4/6 inhibitors may enhance the susceptibility of such tumors to immune checkpoint blockade (53).

### Immune Checkpoint Inhibitors

Immune checkpoint blockade is a promising drug working by blocking checkpoint proteins from binding with their partner proteins. In this review, we will focus on three representative ICBs, PD-1/PD-L1 inhibitor, and CTLA-4 blockage. The effect of ICB on BC patients is still under investigation. However, there are several ongoing trials using PD-1/PD-L1 inhibition and/or CTLA-4 blockage in combination with standard anti-HER2 therapy for HER2+ BC—for example, the phase II DIAmOND study is investigating the combination of PD-L1 and CTLA-4 inhibition added to trastuzumab in patients with HER2+ mBC who progressed on prior trastuzumab-based therapy (168). In another trial, Santa-Maria *et al.* found that the response rates to PD-1/PD-L1 and CTLA-4 inhibition were low in all MBC. However, high rates of clinical benefit were observed in TNBC (169) because of their high expression of these IC molecules. To date, most studies revealed the effect of ICBs on T effector cells, and little is known about their effect on Tregs. As mentioned above, Tconv cells and Treg cells in TME similarly express immune checkpoint molecules, including CTLA-4 and PD-1, at levels that are dependent on the TME, indicating that antibodies targeting these proteins could affect both cell types.

The anti-tumor activity of the anti-CTLA-4 blockade was originally hypothesized to depend on the reinvigoration of dysfunctional CTLA-4-expressing Tconv cells (170). However, evidence from several preclinical studies indicate that the anti-tumor effects of these drugs depend on macrophages depleting Treg cells expressing CTLA-4 in the TME through ADCC, thereby increasing the CTL/Treg cell ratio (62, 98, 99), which implies that CTLA-4 blockade can activate anti-tumor immunity in the presence of enough TILs (171). Nonetheless, there is an absence of studies of Treg depletion in BC TME. Thus, further analyses to address the roles of CTLA-4 in Treg cells in BC settings are warranted.

PD-1 inhibits the excessive activation of Tconv cells by suppression of TCR and costimulatory and renders them dysfunctional or exhausted (172–174). As indicated above, Treg and Tconv cells in the TME express comparable PD-1 and are dependent on TCR and CD28 signaling for their survival and function. PD-1 inhibition potentiates the activation and immunosuppressive function of Treg cells. In line with this hypothesis, a study using a mouse model of autoimmune pancreatitis revealed that PD-1-deficient Treg cells had an increased immunosuppressive activity that was sufficient to rescue the auto-immune phenotype, indicating that PD-1 reduces the immunosuppressive function of Tregs (58). Y Togashi *et al.* found that, *in vitro*, anti-PD-1 mAbs enhance Treg cell-mediated immunosuppression using human samples (175). One of the representative anti-PD1, pembrolizumab, effectively blocked PD-1 expression but did not affect the expression of other Treg-related markers. These results suggest that anti-PD-1 mAbs may reverse immune escape by directly blocking the PD-1/PD-L1 interaction instead of altering the Treg phenotype or function (176).

### Anthracycline-Based Neoadjuvant Chemotherapy

Anthracycline-based neoadjuvant chemotherapy (NAC) with or without taxanes for the initial treatment of patients with invasive BC is the top preoperative systemic therapy regimen recommended by the National Comprehensive Cancer Network panel. In general, the abundance of TILs in BC TME predicts the response to NAC (177, 178). Moreover, Denkert et al. found that the decreased Treg in TME is also linked to the pCR to NAC (179). However, the correlation between pCR and Tregs before NAC is still controversial. Fangxuan Li et al. (180) found that it has no significant relation with pCR. Nevertheless, in some studies, pCR to NAC is associated with less Treg abundance in TNBC but not in ER-positive/Her2-negative breast cancer (79). To be more specific, Ladoire et al. (181) and Senovilla et al. (182) found that, in patients treated with NAC, it is the increased CTL/Treg cell ratio in TME that can precisely predict pCR. Interestingly, the levels of CD8+ T cells and Tregs decreased during NAC in patients of TNBC (183), which raise a question of whether the dynamics of Treg can predict pCR. Hamy et al. (179) found that the decrease of lymphocyte infiltration during chemotherapy is related to the increase of PCR rate, which may be related to the disappearance of Treg after neoadjuvant therapy, but there are few related studies. Adriamycin is one of the typical anthracycline drugs. In BC, docetaxel can indirectly favor immunosurveillance upon polyploidization (182). Moreover, docetaxel is correlated with a reduced activity of Treg in BC and increases the CTL/Treg cell ratio (184). Nevertheless, little studies are conducted to investigate the influence of docetaxel on BC TME.

Anthracycline-based NAC not only contains anthracycline but also can be added with a series of other cytotoxic agents, including taxanes, platinum, and cyclophosphamide (CTK). These cytotoxic treatments can temporarily overcome the immunosuppressive TME, contributing to greater antitumor immune responses (185). CTK embraces direct alkylating and antiangiogenic properties. It is also reported to modulate the immune system in the host through many mechanisms (186). Sistigu et al. (187) reviewed some of these mechanisms, including Th2/Th1 to Th17 shifts in cytokine production, induction of Th17 cells, enhancement of T cell proliferation, resetting of dendritic cell homeostasis, and, more importantly, inhibition of Tregs. However, depending on the dose administered, the antitumor effects of cyclophosphamide can be either through immunopotentiation or direct cytolytic activity (188). Low-dose CTK contributes to antitumor immunity, whereas high-dose CTK works solely through its cytotoxic effects. Patients with breast cancer and treated with metronomic low-dose CTK were found to have a transient reduction in circulating Tregs, lasting 4 to 6 weeks, and diminished functionality (189). Ghiringhelli et al. also found that low-dose CTK depletes Treg cells in peripheral blood, causing the activation of antitumor immunity (190), and thus patients gained survival benefits more or less. However, low-dose CTK also gives rise to higher lymphocyte-infiltrating BC TME, including Treg, but the repletion of Treg cells abolished the antitumor effect of low-dose CTK to some extent (191), which was consistent with a murine experiment (192). These opposite effects of low-dose CTK on circulating and BC TME Treg beg a question on whether low-dose CTK induces the recruitment of Treg from peripheral blood to the TME. In addition to cyclophosphamide, several studieshave revealedthat other cytotoxic agents can also deplete Treg cells. Nevertheless, these data remain controversial, and further preclinical and clinical studies are needed.

### Anti-HER2

HER2-blocking therapies, such as trastuzumab, an IgG1 monoclonal antibody, and/or pertuzumab in combination with chemotherapy, represent the standard first-line treatment for HER2+ BC. In addition to the direct targeting effects on HER2-positive cells, it has been reported that trastuzumab is able to induce a long-lasting immune response in patients with BC (193), but it is still unclear whether trastuzumab has direct effects on Treg immune subsets. A significant decrease in the number of circulating Treg was revealed in patients treated with transtuzumab (194–196). In addition, the decrease of circulating Treg was associated with an objective clinical response or disease stabilization in patients treated with trastuzumab, and the frequency of Treg increased as the disease progressed during trastuzumab treatment (196). Moreover, the recurrence of BC during trastuzumab therapy highly correlates with an increase in Treg frequency. Taken together, circulating Treg can be a predictive marker for response to trastuzumab of the patients.

Small-molecule tyrosine kinase inhibitor (TKIs) is another highly rational anti-HER2 therapeutic regime targeting the adenosine triphosphate (ATP) binding domains of EGFR family due to the homological structure of the ATP, resulting in inhibiting tyrosine kinase phosphorylation (197). It has achieved extreme success in the treatment of other oncogene-driven malignancies. However, treating HER2-positive BC have fallen short of expectations. Some combination therapies of TKIs showed a higher disease-free survival in HER2+ metastatic breast cancer patients (198, 199). Unfortunately, the outcomes of these studies have been disappointing so far. Classic TKIs, such as a dual HER1/HER2 kinase inhibitor, the HER2/HER3 dimerization inhibitor pertuzumab, and the pan-HER (HER1, 2, and 4) kinase inhibitor neratinib can postpone or overcome anti-HER2 resistance and have yielded clinical advantages combined with chemotherapy, hormone therapy, and/or another HER2-inhibiting agent (200, 201). Unlike pertuzumab only improving the anti-trophic effect of the HER2- block, it was shown by the EGF104900 study, lapatinib also amplifies the trastuzumab-induced ADCC effect (202), indicating that lapatinib is more likely to have an antitumor effect through the depletion of Tregs in TME. Additionally, studies from L Hannesdottir et al. (203) in MMTV-neu animals shed light on the effects of lapatinib on enhancing the antitumor immunity. In the neoadjuvant phase II SOLTI-1114 PAMELA trial (NCT01973660), 151 HER2+ BC patients received lapatinib and trastuzumab, plus hormonal therapy if HR+; no significant difference in immune subpopulation densities in TME was observed. BC treated with trastuzumab or/and lapatinib achieving a pCR showed numerically higher densities of Treg cells (204), which is in accordance with the work of Hannesdottir.

## Conclusions

With the deepening of research on TME in breast carcinoma, analysis on the composition of TME becomes increasingly important for evaluating the prognosis of patients with BC disease and the efficacy of different regimes. As a crucial role in TME, the function of Tregs directly and indirectly suppress the anti-tumor immunity through a variety of cellular interactions. In TME, tTreg and pTreg are recruited through the binding of some certain chemokines and their receptors. However, they cannot be easily distinguished. In BC, Tregs have a significantly distinct prognostic value of BC with different subtypes, and the conclusions of these articles are fairly conflicting with each other. By comparing different theories, Tregs are more likely to be an unfavorable factor of the prognosis of BC as a whole. However, further research or meta-analysis needs to be done to verify this effect. In view of the discovery of the great potential value of Treg, Treg cells are under intense scientific and commercial scrutiny as a novel therapeutic strategy or biomarker for anticancer treatment. Some classic regimes, such as anthracycline-based NAC, anti-Her2 treatment, immune checkpoint inhibitor, and cyclin-dependent kinases 4 and 6 (CDK4/6), proved to have a strong impact on depleting Treg in BC TME through different immunological effects. The link between Treg and the efficacy evaluation of tumor response to different treatments is found in anthracycline-based NAC, anti-HER2 NAC, but the relationship is still unknown in other treatments, which is a potential research field for us to manipulate Treg to reach the highest efficacy of these treatment strategies.

## Author Contributions

JL wrote the manuscript. XW contributed significantly to manuscript preparation. YD and XY conceived the structure and revised the manuscript. HW complemented relevant content and revised illustration and ZL revised the manuscript. All authors contributed to the article and approved the submitted version.

## Funding

This study was supported by Haiyan Foundation of Harbin Medical University (JJMS2020-04) and National Natural Science Foundation of China (Nos. 82073146).

## Acknowledgment

We would like show sincere appreciation to the constructive suggestion provided by our colleagues in Department of Surgical Oncology.

## Conflict of Interest

The authors declare that the research was conducted in the absence of any commercial or financial relationships that could be construed as a potential conflict of interest.

## Publisher’s Note

All claims expressed in this article are solely those of the authors and do not necessarily represent those of their affiliated organizations, or those of the publisher, the editors and the reviewers. Any product that may be evaluated in this article, or claim that may be made by its manufacturer, is not guaranteed or endorsed by the publisher.

## Abbreviations

MHC, major histocompatibility complex; CTLs, cytotoxic T lymphocyte; TME, tumor microenvironment; Treg, regulatory T cell; BC, breast cancer; TILs, tumor-infiltrating lymphocytes; OS, overall survival; APCs, antigen-presenting cells; TCR, T cell receptor; JAK, Janus kinase; PI3K, phosphoinositide 3-kinase; MAPK, mitogen-activated protein kinase; STAT5, signal transducer and activator of transcription 5; mTORC2, mTOR complex 2; FOXO, Forkhead box O; tTreg, thymus-derived Treg; pTreg, peripheral Treg; Tconv, conventional T cells; TCGA, The Cancer Genome Atlas; AJCC, American Joint Committee on Cancer; Th1, T helper type 1; Th2, T helper type 2; CNA, copy number alteration; BCSS, breast cancer specific survival; pCR, pathological completed response; ORR, objective response rate; NAC, neoadjuvant chemotherapy; CTK, cyclophosphamide; TKIs, tyrosine kinase inhibitors; ADCC, antibody-dependent cellular cytotoxicity; CDK4/6, cyclin-dependent kinases 4 and 6; DNMT1, DNA methyltransferase 1; TGF- β, transforming growth factor-β; IL-2R, interlukin-2 receptor.
